# Genomic characterization of a pectinolytic isolate of *Serratia oryzae* isolated from lake water

**DOI:** 10.7150/jgen.38365

**Published:** 2019-10-15

**Authors:** Nicole Hugouvieux-Cotte-Pattat, Cécile Jacot-des-Combes, Jérôme Briolay

**Affiliations:** 1 Univ Lyon, CNRS, INSA Lyon, Université Claude Bernard Lyon 1, UMR5240 Microbiologie Adaptation et Pathogénie, F-69621Villeurbanne, France; 2 Univ Lyon, Université Claude Bernard Lyon 1, CNRS, plateforme DTAMB, FR3728 BioEnviS, F-69621Villeurbanne, France

**Keywords:** *Serratia oryzae*, isolate, lake water

## Abstract

Only one isolate of* Serratia oryzae*, the type strain J11-6^T^ has been characterized up to now. This strain was found in the endophytic bacterial flora of rice. As part of an ongoing investigation into pectinolytic bacteria present in lake water in France, a few *Serratia* strains were isolated, including S32 and J9 identified as new strains of *S. oryzae*. The genome of strain S32 consists of a circular chromosome of 4,810,389 bp that contains 4,584 protein-coding genes. The genome of S32, as well as those of the type strain J11-6^T^, contains several genes involved in pectin degradation and in the intracellular assimilation of pectin oligomers. The specific detection of enzyme activities confirmed that strain S32 secretes functional pectinases and that it also produces extracellular cellulase and protease activities. The ability to produce plant cell wall degrading enzymes shows that *S. oryzae* shares characteristics of plant associated bacteria, including phytopathogens.

## Introduction

The *Serratia* genus is a member of the *Yersiniaceae* family, in the *Enterobacterales* order 1*.* This family comprises seven genera *Chania, Ewingella, Rahnella, Rouxiella, Samsonia, Serratia*, and *Yersinia* 1. Bacteria of the genus *Serratia* are common in the environment and were isolated from a variety of niches, including soil, water, air, plants, and animals 2. Some species, such as *S. rubidiae*, *S. ficaria* and *S. oryzae* are closely associated with plants 3, 4, 5. Other species, such as *S. fonticola* and* S. aquatilis,* occur naturally in aquatic environments 6, 7.

Our laboratory is interested in bacteria of the genus *Dickeya*, an important group of plant pathogens 8. In the order* Enterobacterales*, *Dickeya* species belong to the family of *Pectobacteriaceae* that includes the five genera *Brenneria*, *Dickeya*,* Lonsdalea*, *Pectobacterium*, and *Sodalis. Dickeya* and *Pectobacterium* species provoke soft-rot diseases on a wide range of hosts, due to the action of extracellular pectinases that attack the plant cell wall 9. They are frequently isolated from vegetable crops or ornamental plants, but also from water. To better understand the natural diversity of *Dickeya* species, we performed a survey of pectinolytic bacteria occurring in the water of small lakes of the French region La Dombes. Strain selection was based on a semi-selective solid medium which includes pectin as a gelling agent; this medium is commonly used for the isolation of *Dickeya* and *Pectobacterium* 10. We found several pectinolytic isolates in lake water, including a new *Dickeya* species, *D. lacustris* 11. However, preliminary genetic analysis indicated that some of the isolates did not belong to the *Pectobacteriaceae* family. For instance, the two isolates S32 and J9 showed a high pectinolytic activity and genetic analysis suggested that they belong to the *Serratia* genus and, most probably, to the species *S. oryzae*. Currently, only one *S. oryzae* strain, the type strain J11-6^T^, has been characterized 5. This strain was found in the endophytic bacterial flora of rice. We sequenced the S32 genome to gain knowledge on the natural variability of *S. oryzae* and in the factors involved in its adaptation to the plant or aquatic environment.

## Methods

### Isolation of pectinolytic bacteria

Strains J9 and S32 were isolated in water from lakes of the wetland region La Dombes in France. The site of Foundation Pierre Vérots (latitude 45.951937 and longitude 4.883082) is reserved for the study and preservation of biodiversity. The anthropogenic contribution is minimal into this site. It contains four lakes surrounded by wet meadows and forests. Water samples were collected from the lakesides approximately 1.5 m from the shore and at less than 0.5 m depth. The water of the lakes is naturally eutrophic and rich in plant debris.

A first filtration on pleated paper filters was used to remove large particles from the eutrophic water. Then, the samples were used directly or concentrated 30x by centrifugation for 10 min at 8 000 g (the pellet of a 15 ml sample was suspended in 0.5 ml of sterile water). Aliquots of 100 μl of water, before and after concentration, were spread onto crystal violet pectate (CVP) plates. After incubating for 48 h at 30°C, pectinolytic bacteria were detected by the formation of cavities around colonies, due to the degradation of polygalacturonic acid 10. Strain J9 was isolated in June 2017 from a water sample of lake Riquet (5 ha) at a temperature of 29.2 °C. Strain S32 was isolated in September 2017 from a concentrated water sample of lake Boufflers (28 ha) at a temperature of 18.6 °C.

### Genomic DNA extraction, genome sequencing and analysis

The total bacterial genomic DNA of strain S32 was extracted using a NucleoSpin^R^ bacterial DNA purification kit (Macherey-Nagel). Quantification and quality control of the DNA was performed using a Nanodrop spectrophotometer, a Qubit4 fluorometer and agarose gel electrophoresis. Genomic DNA was sequenced using a MiSeq Illumina platform (Biofidal France). RAST was used for automatic annotation of the genome 12. This annotation was used to search for genes potentially involved in pectin catabolism. Polysaccharide lyases (PLs) and glycoside hydrolases (GHs) were classified according to CAZY (http://www.cazy.org/). Blast comparisons were carried out using the NCBI website (https://blast.ncbi.nlm.nih.gov/Blast.cgi). The prediction of signal peptides was performed using SignalP 5.0 (http://www.cbs.dtu.dk/services/SignalP/).

PCR amplifications were performed on bacterial cell lysates using an Illustra^TM^ PuReTaq^TM^ Ready-To-Go^TM^ kit (GE Healthcare). To perform a preliminary strain identification, PCR amplification was performed using the primers gapA-7-F and gapA-938-R 13 and the *gapA* amplicon sequences were determined by Sanger sequencing (Biofidal France). The strain taxonomic position was further clarified by calculation of average nucleotide identity (ANI) values (http://enve-omics.ce.gatech.edu/ani/) 14. These analyses were performed using the genomes of a selection of 24 well-characterized *Serratia* strains representative of 13 species and including 11 type strains.

Phylogenetic trees were constructed using the neighbour-joining method 15, with calculation of the percentages of replicate trees in which the associated taxa clustered together in the bootstrap test (1000 replicates) 16. For DNA, the evolutionary distances (number of base substitutions per site) were computed using the maximum composite likelihood method 17. For proteins, the evolutionary distances were computed using the Poisson correction method 18. Evolutionary analyses were conducted in MEGA7 19.

### Phenotypic characterization

To test their growth with different carbon sources, strains were inoculated onto M63 minimal medium plates supplemented with the appropriate carbon source (2 g l^-1^). Growth was recorded after incubating for 24 and 48 h at 30°C.

Production of extracellular enzymes was detected on specific media. Pectinase secretion was detected after growth for 24 h at 30°C on plates containing M63 medium supplemented with glycerol (2 g l^-1^) and polygalacturonate (4 g l^-1^). After flooding the plates with a saturated solution of copper acetate, clear zones appear around the colonies able to degrade polygalacturonate 20. Cellulase secretion was detected after growth for 24 h at 30°C on plates containing M63 medium supplemented with glycerol (2 g l^-1^) and carboxy-methyl-cellulose (10 g l^-1^). After flooding with congo red (5 g l^-1^) and washing with 1 M NaCl, clear zones appear around the colonies able to degrade cellulose 20. Protease secretion was tested on LB agar plates containing skim milk powder (6.25 g l^-1^), and clear zones around colonies were recorded after plate incubation for 24 and 48 h at 30°C 20. Lecithinase secretion was tested on LB agar plates containing 5% egg yolk, and opalescent zones around colonies were recorded after plate incubation for 24 and 48 h at 30°C 21. DNase secretion was tested on M63 agar plates containing salmon sperm DNA (1 g l^-1^) and methyl green (0.2 g l^-1^); light-green zones around colonies were recorded after plate incubation for 48 h at 30°C 22.

### Bacterial motility and maceration ability

The swimming motility, inside the soft agar medium, was estimated by pricking 3 bacterial colonies with a thin rod inside a 0.3% GL agar plate which was then incubated at 30°C for 24 h before measuring the growth diameter.

The swarming motility, at the surface of the agar medium, was estimated by inoculating 3 drops of 2 μl of a fresh bacterial culture on a 0.6% GL agar plate supplemented with glucose (5 g l^-1^). The plates were incubated at 30°C for 24 h before measuring colony expansion. Surfactant production was visualized by the presence of a transparent film on the plate surface around the growth zone.

To test the maceration capacity on plant tissues, we inoculated chicory leaves and potato tubers 23. Seven chicory leaves were infected for each strain, using 10 µl of bacterial suspension (10^9^ cfu/ml in M63 medium). After incubation in a humid chamber for 24 h at 30°C, the length of macerated tissue was measured. Ten potato tubers were inoculated with 5 µl of bacterial suspension (10^9^ cfu/ml in M63 medium). Following incubation for 48 h at 30°C in a humid chamber, the weight of rotten tissue was measured to estimate the maceration ability. The model *Dickeya* strai*n, D. dadantii* 3937, was used as a positive control in these experiments. Negative controls were performed using M63 medium.

## Results and Discussion

The high pectinolytic activity of the two isolates S32 and J9 justified their careful identification. As a first approach, their *gapA* gene was amplified by PCR and sequenced. Analysis of the *gapA* amplicons showed a close proximity between strains J9 and S32 (99% identity). Blast search against nr/nt NCBI databank indicated that the best scores (94 to 95% identities) for the J9 or S32 *gapA* amplicons are found with strains of the genus *Serratia,* including several strains of* S. fonticola, S. liquefaciens, S. proteamaculans, S. quinivorans,* and of non-identified *Serratia* species. The level of identity with strains of other characterized *Serratia* species is lower than 90%. To gain information on this water isolate, the genome sequence of strain S32 was determined. The draft genome comprises 149 contigs (N50=119,595, L50=14, 324X coverage depth) with a total length of 4,810,389 bp and a G+C content (mol%) of 53.3%. The S32 genome was automatically annotated with RAST 12, predicting 4,584 protein coding genes and 84 RNA-coding sequences, including 75 tRNAs and 9 rRNAs (23S, 16S, and 7 x 5S).

**Figure 1:**
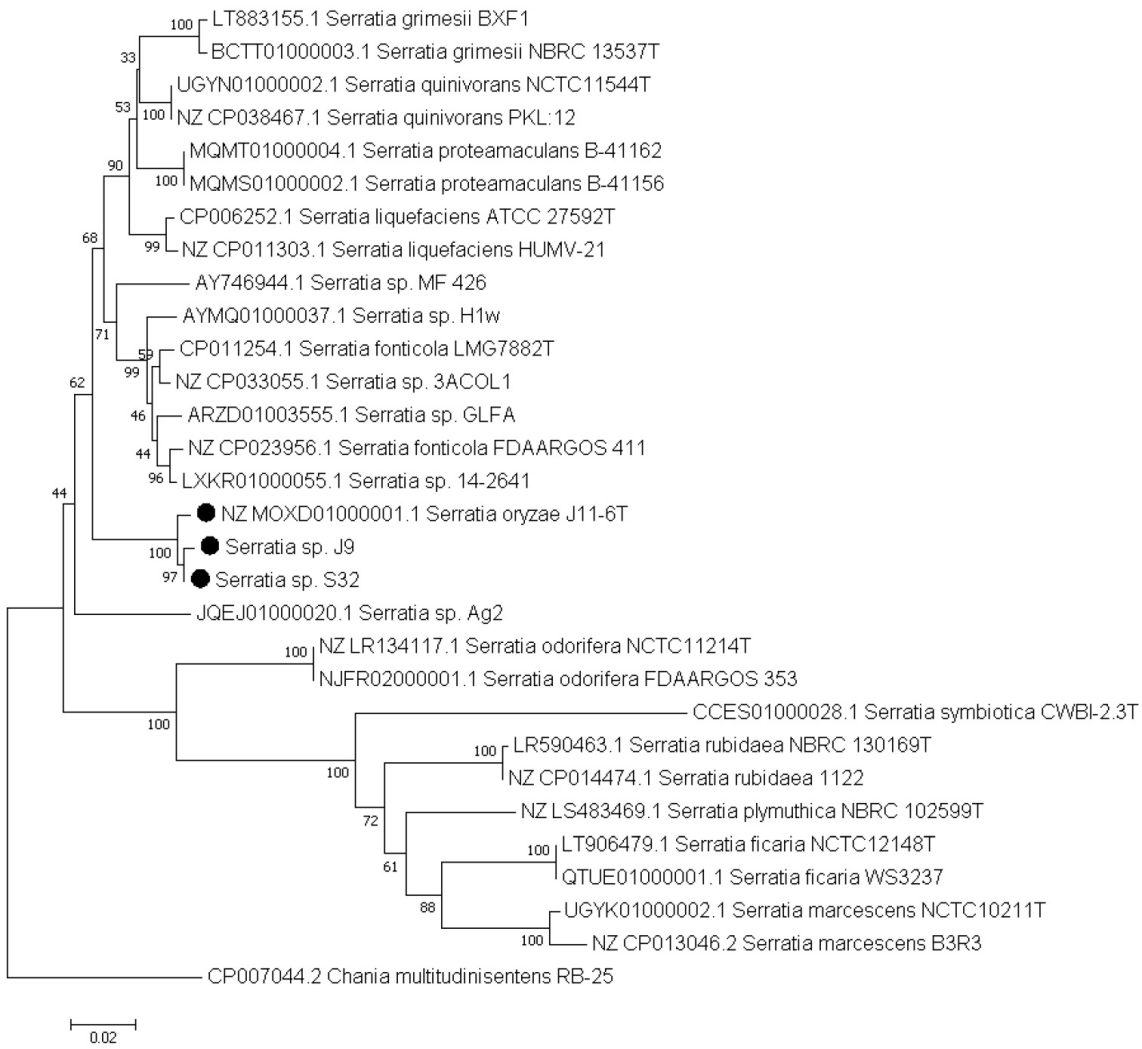
**Phylogenetic position of strain S32 and J9 based on the *gapA* sequences.** This analysis was performed using the *gapA* sequences of strains belonging to well-characterized *Serratia* species, and of *Serratia* sp. strains whose genome encodes a PL1 pectate lyase. The gene *gapA* of *Chania multitudinisentens* was used as an outgroup. The evolutionary history was inferred using the neighbour-joining method 15. The percentages of replicate trees in which the associated taxa clustered together in the bootstrap test are shown next to the branches 16. The evolutionary distances were computed using the maximum composite likelihood method 17 and are in the units of the number of base substitutions per site (718 positions). Evolutionary analyses were conducted in MEGA7 19.

The most relevant parameter for ascertaining the identity of a strain is the calculation of ANI that measure the genetic distance between two genomes 14. The taxonomic identification of strain S32 was clarified by calculation of ANI values based on pairwise comparisons between the S32 genome and a selection of 24 genomes of characterized* Serratia* species. An ANI value higher than 99% was found with *S. oryzae* J11-6^T^ and ANI values of 81-83% were observed with other *Serratia* species (Table 1). According to a threshold of 95% for delineating the species boundaries 14, S32 belongs to the species *S. oryzae*. A new Blast search against the J11-6^T^ genome (NCBI assembly GCA_001976145.1) using the sequence of the S32 or J9 *gapA* amplicons showed that each *gapA* sequence shares more than 99% identity with J11-6^T^ DNA, confirming the close proximity between the three strains (Figure 1).

A high level of homology was found between the genome of S32 and that of the type strain of* S. oryzae*, J11-6^T^. The complete genome sequence of *S. oryzae* J11-6^T^ consists of 5,012,778 bp, with G+C contents of 53.2% and 4,733 predicted protein coding genes by automatic annotation with RAST. Using the function-based comparison tool in RAST, 88 genes were predicted to be present in S32 but absent in J11-6^T^ and, inversely, 64 genes were predicted in J11-6^T^ but absent in S32. Several S32 specific genes are related to phages or mobile elements (25 genes), or predicted to encode fimbriae (5 genes), toxins/antitoxins (5 genes), or restriction-modification systems (5 genes) (Table S1). Several genes present in J11-6^T^ but absent in S32 also correspond to phages or mobile elements (21 genes); others are predicted to encode proteins involved in adhesion (11 genes), urease subunits (8 genes), or CRISPR- associated proteins (7 genes) (Table S2). Such strains specific genes often occur in clusters, suggesting horizontal acquisition.

**Figure 2:**
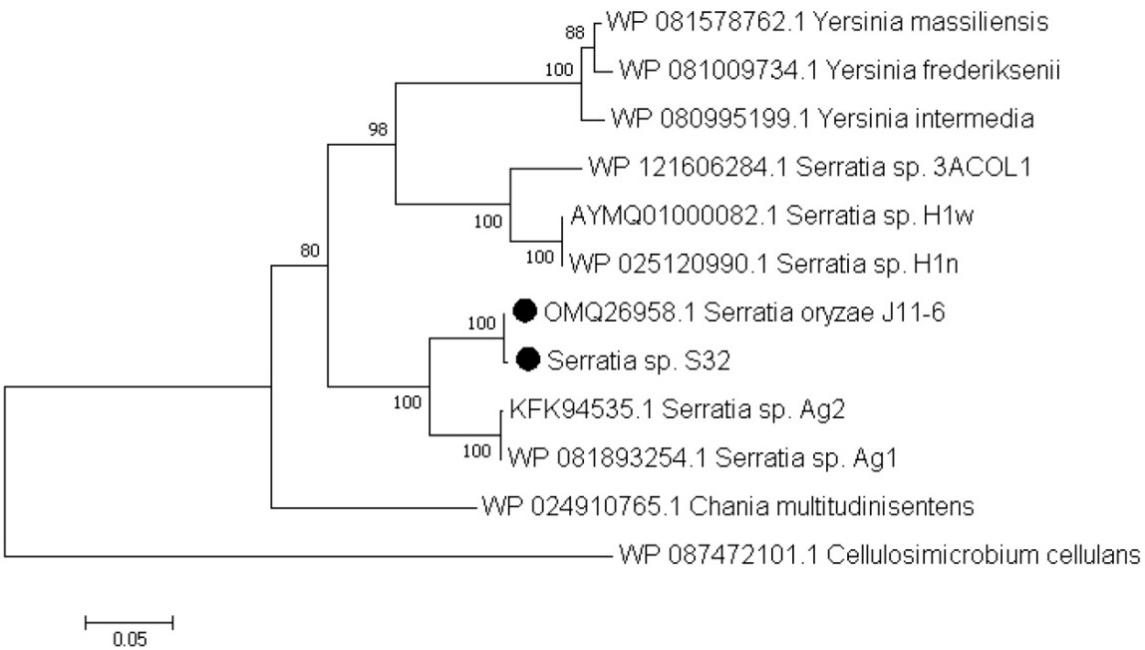
**Phylogenetic tree of the PL1 protein Pel1 of strain S32.** The evolutionary history was inferred using the neighbour-joining method 15. The percentages of replicate trees in which the associated taxa clustered together in the bootstrap test (1000 replicates) are shown next to the branches 16. The evolutionary distances were computed using the Poisson correction method 18 and are in the units of the number of amino acid substitutions per site (430 positions). Evolutionary analyses were conducted in MEGA7 19. This analysis was performed using the sequences of the different *Serratia* strains encoding a PL1 protein. A homologous protein of *Cellulosimicrobium cellulans* was used as an outgroup.

Based on the RAST annotations, we searched for genes potentially involved in pectin catabolism. Similar genes were found in both S32 and J11-6^T^ genomes, reflecting their ability in degrading this plant polysaccharide. Both strains contain genes encoding a predicted pectate lyase of the family PL1, three putative polygalacturonases of the family GH28 and an oligogalacturonate lyase of the family PL22. Among the sequenced *Serratia* strains, a few other genomes contain potential pectinase genes (http://www.cazy.org/). Some *S. fonticola* and *S. odorifera* genomes are predicted to encode a PL9 pectate lyase, a PL22 oligogalacturonate lyase and one or two GH28 polygalacturonases. However, among the sequenced *Serratia* strains, proteins of the family PL1 are rare and found only in *S. oryzae* J11-6^T^ and a few strains of non-characterized species (Figure 2). In contrast, in living organisms, PL1 is the largest family of pectate lyases, including several hundred representatives from different kingdoms, plants, fungi, oomycetes, bacteria and archaea 24. Plant-pathogenic bacteria often possess several PL1 members which have a high specific activity. For instance, the phytopathogen *D. dadantii* 3937 encodes eight PL1 proteins, including six characterized pectate lyases and two putative pectin lyases 24. The PL1 pectate-lyase of S32, Pel1, shares homology with proteins of *S. oryzae* J11-6 (99.8% identity), five non-identified *Serratia* strains (Ag1, Ag2, H1n, H1w, and 3ACOL1,with 91, 91, 78, 78 and 76% identity, respectively), *Chania multitudinisentens* (79% identity) and diverse strains of three *Yersinia* species, *Y. massiliensis, Y. frederiksenii* and *Y. intermedia* (about 75% identity) (Figure 2). Outside these proteins from *Enterobacterales*, several Pel1 homologs are found in Gram positive bacteria such as *Cellulosimicrobium* and *Cellulomonas* (about 53 and 51 % identity, respectively). None of these bacteria encoding a Pel1 homolog has been previously reported to be a phytopathogen. Comparison with proteins encoded by *D. dadantii* 3937 showed that the *S. oryzae* Pel1 shares about 30% identity with the major pectate lyases PelA, PelB, PelC, PelD and PelE of this plant pathogenic species 24.

The pectate-lyase Pel1 of S32 presents a N-terminal signal peptide typical of precursor proteins transported by the Sec translocon and cleaved by Signal Peptidase I 25, suggesting its exportation in the bacterial periplasm. Enzymatic assays demonstrated the presence in the S32 culture supernatant of a pectate lyase activity able to cleave polygalacturonate, the pectin backbone. A gene cluster encoding a complete type II secretion system (T2SS) is present in *S. oryzae* genome; it could be involved in Pel1 secretion. T2SS mediates secretion of proteins from the periplasm to the external medium and a well-studied T2SS is responsible for specific pectate lyase secretion in the genus *Dickeya* 26. Two GH 28 polygalacturonases of S32 present the N-terminal lipoprotein signal peptide typical of proteins transported by the Sec translocon and cleaved by Signal Peptidase II 25. Thus, these two proteins are predicted to be anchored to the bacterial outer membrane. Some *D. dadantii* pectinases have already been shown to be associated with the outer membrane, anchored either on the periplasmic or extracellular sides of this membrane 27, 28.

**Figure 3:**
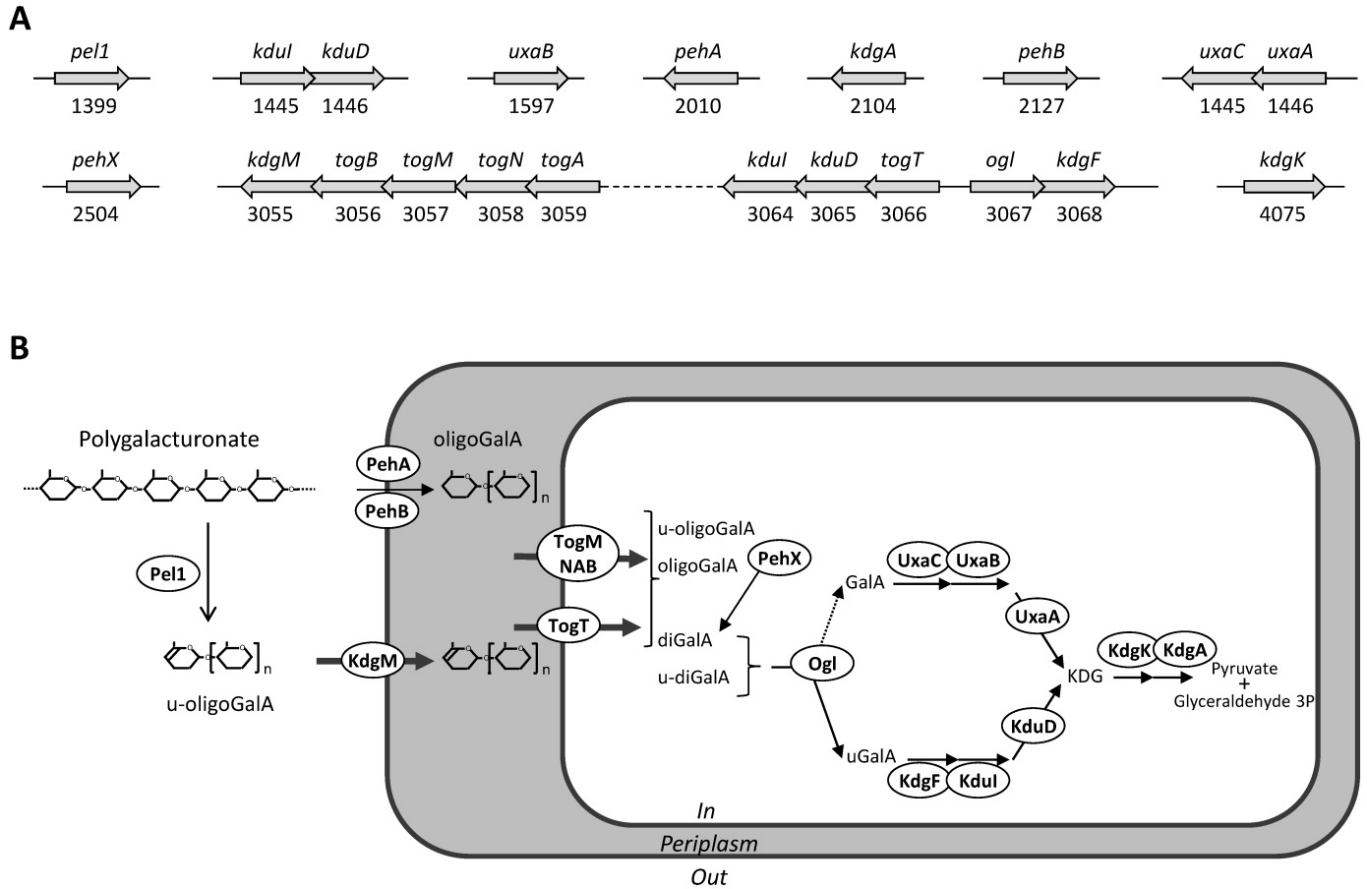
**Genes and proteins of strain S32 potentially involved in pectin catabolism. A:** Genetic organization. Genes are symbolized by arrows; their name and ID number are given. **B:** Proposed metabolic pathway deduced from the predicted protein localization and from the function of homologous proteins. The extracellularly pectate lyase Pel1 generates unsaturated oligogalacturonides (u-oligoGalA) up to dimers (u-diGalA, unsaturated digalacturonide). The activity of outer membrane anchored polygalacturonases, PehA and PehB, generates saturated oligomers up to dimers (oligoGalA, oligogalacturonides; diGalA, digalacturonide). Extracellular oligomers enter the periplasm using the specific outer-membrane porin, KdgM. Short oligomers enter the cytoplasm using two specific transporters, TogMNAB and TogT. In the cytoplasm, they are further cleaved up monomers by the action of the exopolygalacturonase PehX and the oligogalacturonate lyase, Ogl. The two pathways involved in the catabolism of galacturonate (GalA) and unsaturated galacturonate (uGalA) converge to produce a common intermediate, KDG (2-keto-3-deoxygluconate) which is converted to pyruvate and glyceraldehyde 3-phosphate.

Both S32 and J11-6^T^ genomes have also all the genes necessary for intracellular pectin metabolism, encoding the transporters KdgM, TogMNAB and TogT 29, and the enzymes KdgF, KduI, KduD, KdgK, and KdgA 30, 31 (Figure 3). All these catabolic steps are also encoded by the *Dickeya* genomes, allowing bacteria to efficiently use oligosaccharides resulting from degradation of the pectin backbone, as a sole carbon and energy source for growth 32.

Diverse compounds were independently added to minimal medium to test their utilization as a sole carbon source by strains S32 and J9. This phenotypic analysis confirms that the two strains are able to grow in the presence of polygalacturonate as a sole carbon source. They are also able to grow in the presence of several monosaccharides or disaccharides, such as D-arabinose, D-fructose, D-galactose, D-galacturonate, D-glucose, D-gluconate, D-glucuronate, m-inositol, D-mannitol, D-mannose, melibiose, L-rhamnose, D-ribose, sucrose, trehalose, and D-xylose. The use of different biochemical tests for the specific detection of extracellular enzyme activities showed that both S32 and J9 secrete pectinase, cellulase and protease activities (Table 2). However, the two strains are not strictly identical; the enzymatic activities of J9 are higher than that of S32, particularly for cellulase and lecithinase production (Table 2). Since they produce extracellular pectate lyase activity, we tested the potential ability of these strains to macerate plant tissues (Table 3). S32 showed a significant ability to macerate plant tissues; the amount of rotten tissue obtained either on chicory leaves or potato tubers, was about one-third that obtained with the plant pathogen, *D. dadantii* (Table 3). Strain S32 exhibited high swimming and swarming motilities and surfactant production (Table 3). Strain J9 showed a lower maceration capacity than S32, probably related to its lower motility (Table 3).

The previously characterized *S. oryzae* type strain, J11-6^T^, was found in the endophytic bacterial flora of rice 5. The two new *S. oryzae* strains S32 and J9 were isolated from water of eutrophic lakes. They showed a high pectinolytic activity and production of plant cell wall degrading enzymes, indicating that *S. oryzae* strains share characteristics of plant associated bacteria, including phytopathogens. Since the two strains S32 and J9 are similar but not identical, it would be interesting to test the phenotypic features of the type strain J11-6^T^ to gain insights in the natural diversity of *S. oryzae* strains. The *S. oryzae* genomes encode an extracellular pectate lyase of the PL1 family. These enzymes play various physiological roles; they are involved in the maceration of plant tissues caused by pectinolytic phytopathogens, in the establishment of symbiosis by rhizobia, in the degradation of plant litter in the soil, or in the digestion of vegetable food by gut bacteria. The *S. oryzae* pectate lyase could allow these bacteria to use pectin as a carbon source for growth and to efficiently degrade plant debris found in their close environment, soil or water.

### Nucleotide sequence accession numbers

This Whole Genome Shotgun project has been deposited at DDBJ/ENA/GenBank under the accession SOZF00000000. The S32 genome draft version described in this paper is version SOZF01000000.

## Supplementary Material

Supplementary figures and tables.

## Figures and Tables

**Table 1:**
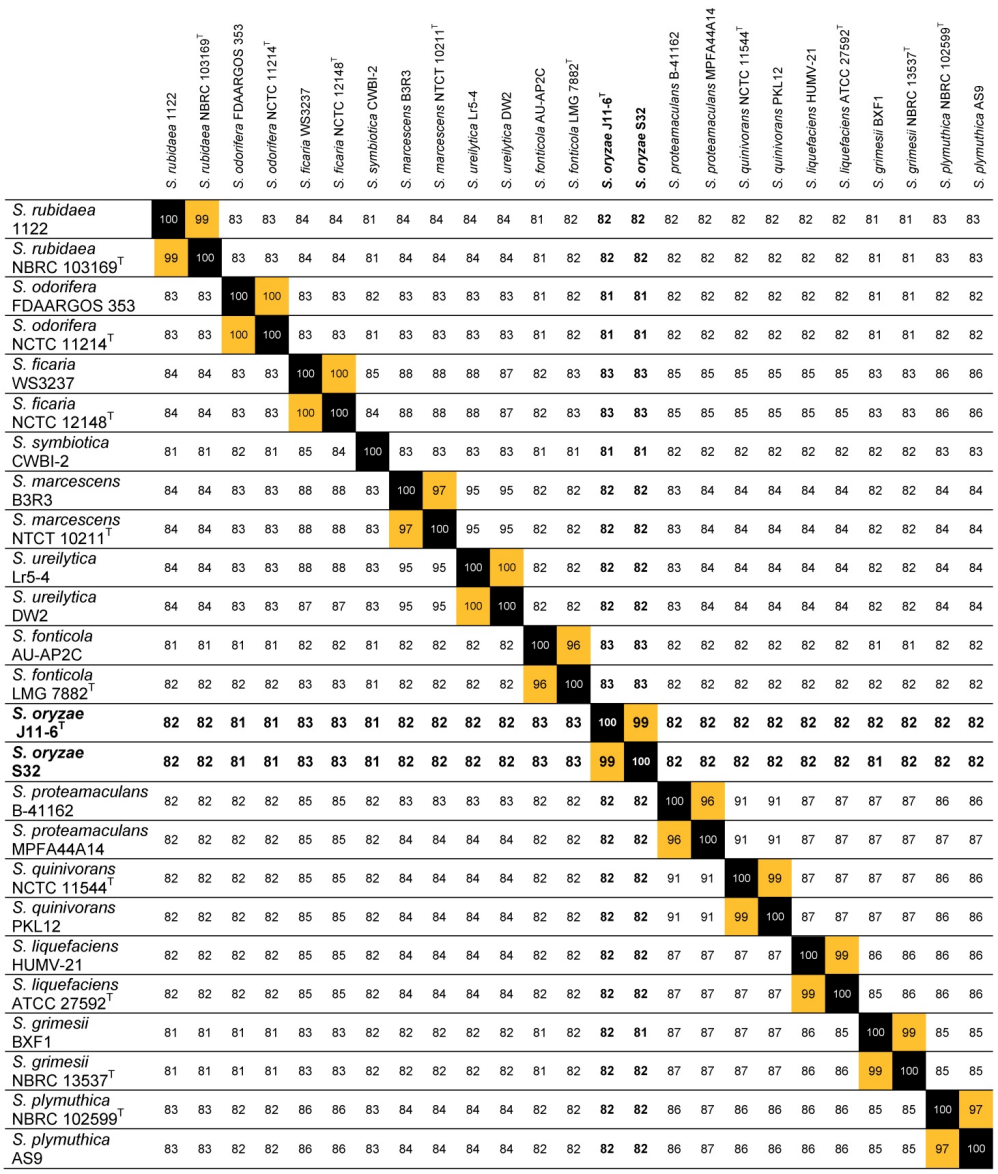
** ANI values between strain S32 and a selection of *Serratia* strains.** This analysis was performed using the genomes of the strains *S. ficaria* WS3237 (NZ_QTUE01000001.1) and NCTC 12148^T^ (LT906479.1), *S. fonticola* AU-AP2C (ASZA01000001.1) and LMG 7882^T^ (AVAH01000001.1), *S. grimesii* BXF1 (LT883155.1) and NBRC 13537^T^ (BCTT01000001.1), *S. liquefaciens* HUMV-21 (CP011303.1) and ATCC 27592^T^ (CP006252.1), *S. marcescens* B3R3 (CP011303.1) and NTCT 10211^T^ (UGYK01000001.1), *S. odorifera* FDAARGOS 353 (NJFR02000001.1) and NCTC 11214^T^ (NZ_LR134117.1), *S. oryzae* J11-6^T^ (MOXD01000001.1), *S. plymuthica* NBRC 102599^T^ (BCTU01000001.1) and AS9 (CP002773.1), *S. proteamaculans* B-41162 (MQMT01000001.1) and MPFA44A14 (FWWG01000001.1), *S. quinivorans* NCTC 11544^T^ (UGYN01000001.1) and PKL12 (CP038467.1), *S. rubidaea* 1122 (CP014474.1) and NBRC 103169^T^ (BCZJ01000001.1), *S. symbiotica* CWBI-2 (CCES01000001.1), *S. ureilytica* Lr5-4 (JSFB01000001.1) and DW2 (PGPC01000001.1).

**Table 2:** Production of extracellular enzymes by *Serratia oryzae. D. dadantii* 3937 was used as a reference strain. (+, positive; -, negative; w, weak)

	Serratia oryzae		Dickeya dadantii
	S32	J9	3937
Pectinase	+	+	+
Cellulase	w	+	+
Protease	+	+	+
Lecithinase	-	w	+
DNase	-	-	+

**Table 3:** ** Maceration ability and motility of *Serratia oryzae.*** To estimate the maceration ability, the length of macerated tissue was measured 24 h after inoculation for chicory leaves and the weight of macerated tissue was measured after 48 h for potato tubers. The mean values are given with the standard deviations. To estimate the bacterial motility, the growth diameter was measured 24 h after inoculation in 0.3% GL agar plate or on 0.6% GL agar plate, respectively. Surfactant production was visualized by a transparent film on the plate surface. *D. dadantii* 3937 was used as a reference strain.

	Serratia oryzae		Dickeya dadantii
	S32	J9	3937
Chicory leaf maceration (mm)	33±9	10±4	98±11
Potato tuber maceration (g)	1.26±0.45	0.02±0.01	3.69±0.68
Swimming motility (mm)	45±2	5±1	29±1
Swarming motility (mm)	35±4	7±1	26±4
Surfactant production	+	-	+

## Abbreviations

ANI: average nucleotide identity
CAZY: Carbohydrate-Active enZYmes
CVP: crystal violet pectate medium
GH: glycoside hydrolase
LB: Luria-Bertani medium
PSL: polysaccharide lyase
RAST: rapid annotations using subsystems technology
T2SS: type II secretion system
