# Supplementary material for: Genomic characterization of a pectinolytic isolate of Serratia oryzae isolated from lake water
Source: J Genomics. 2019 Oct 15;7:64–72. doi: 10.7150/jgen.38365 (PMC6831795; doi:10.7150/jgen.38365)
Supplement: Supplementary file 1 — Supplementary figures and tables. [file jgenv07p0064s1.pdf]

Table S1. S32 genes that are absent in strain J11-6^{T}

Gene id	Length (aa)	Predicted function
fig|2034155.4.peg.2	173	UDP-galactopyranose mutase (EC 5.4.99.9)
fig|2034155.4.peg.3	168	Succinate dehydrogenase flavoprotein subunit (EC 1.3.5.1)
fig|2034155.4.peg.7	162	Thymidine phosphorylase (EC 2.4.2.4)
fig|2034155.4.peg.243	723	FIG035595: Phage protein
fig|2034155.4.peg.298	711	TonB-dependent receptor
fig|2034155.4.peg.302	83	Programmed cell death antitoxin MazE
fig|2034155.4.peg.303	111	Programmed cell death toxin MazF
fig|2034155.4.peg.504	423	Mobile element protein
fig|2034155.4.peg.505	1086	Type I restriction-modification system, restriction subunit R (EC 3.1.21.3)
fig|2034155.4.peg.506	436	Type I restriction-modification system, specificity subunit S
fig|2034155.4.peg.516	175	phage-associated protein
fig|2034155.4.peg.520	193	Uncharacterized protein YmfQ in lambdoid prophage e14 region
fig|2034155.4.peg.521	353	Putative protein JayE from lambdoid prophage e14 region
fig|2034155.4.peg.522	138	Bacteriophage protein GP46
fig|2034155.4.peg.523	180	Prophage baseplate assembly protein V
fig|2034155.4.peg.524	360	FIG003269: Prophage tail protein
fig|2034155.4.peg.525	353	Phage tail/DNA circulation protein
fig|2034155.4.peg.527	90	L33
fig|2034155.4.peg.533	343	Adenine-specific methyltransferase (EC 2.1.1.72)
fig|2034155.4.peg.1773	82	Phage replication protein
fig|2034155.4.peg.1951	194	hypothetical protein; Some similarities with unknown protein
fig|2034155.4.peg.1953	81	hypothetical protein; Some similarities with unknown protein
fig|2034155.4.peg.2016	102	HipB protein @ Antitoxin HigA
fig|2034155.4.peg.2017	445	Toxin HigB / Protein kinase domain of HipA
fig|2034155.4.peg.2023	303	Retron-type RNA-directed DNA polymerase (EC 2.7.7.49)
fig|2034155.4.peg.2025	96	Transcriptional regulator
fig|2034155.4.peg.2026	101	FIG022160: hypothetical toxin
fig|2034155.4.peg.2041	373	Lipid carrier: UDP-N-acetylgalactosaminyltransferase (EC 2.4.1.-); Putative glycosyltransferase
fig|2034155.4.peg.2042	140	FIG01210424: hypothetical protein
fig|2034155.4.peg.3173	1153	Phage tail fiber protein
fig|2034155.4.peg.3174	207	Phage tail assembly protein I
fig|2034155.4.peg.3175	106	Phage tail assembly protein
fig|2034155.4.peg.3176	165	Phage integrase
fig|2034155.4.peg.3267	160	COG0286: Type I restriction-modification system methyltransferase subunit
fig|2034155.4.peg.3300	180	Lead, cadmium, zinc and mercury transporting ATPase (EC 3.6.3.3) (EC 3.6.3.5); Copper-translocating P-type ATPase (EC 3.6.3.4)
fig|2034155.4.peg.3307	337	DNA-cytosine methyltransferase (EC 2.1.1.37)
fig|2034155.4.peg.3310	659	Type III restriction-modification system methylation subunit (EC 2.1.1.72)
fig|2034155.4.peg.3311	987	Type III restriction-modification system restriction subunit (EC 3.1.21.5)
fig|2034155.4.peg.3313	301	Chromosome (plasmid) partitioning protein ParB
fig|2034155.4.peg.3314	511	Recombinase
fig|2034155.4.peg.3317	497	hypothetical protein; Some similarities with nicking enzyme (TraA)
fig|2034155.4.peg.3582	164	Transposase
fig|2034155.4.peg.3588	198	CFA/I fimbrial major subunit

fig|2034155.4.peg.3589	232	CFA/I fimbrial auxiliary subunit
fig|2034155.4.peg.3590	833	CFA/I fimbrial subunit C usher protein
fig|2034155.4.peg.3591	530	CFA/I fimbrial minor adhesin
fig|2034155.4.peg.3592	225	CFA/I fimbrial chaperone
fig|2034155.4.peg.3602	73	Mobile element protein
fig|2034155.4.peg.3603	216	Metal-binding protein ZinT
fig|2034155.4.peg.3608	353	Uncharacterized SAM-dependent O-methyltransferase
fig|2034155.4.peg.3609	241	3-oxoacyl-[ACP] synthase
fig|2034155.4.peg.3610	264	FIG018329: 1-acyl-sn-glycerol-3-phosphate acyltransferase
fig|2034155.4.peg.3611	86	Acyl carrier protein (ACP1)
fig|2034155.4.peg.3612	84	Acyl carrier protein (ACP2)
fig|2034155.4.peg.3613	194	FIG017861: hypothetical protein
fig|2034155.4.peg.3614	467	FIGfam138462: Acyl-CoA synthetase, AMP-(fatty) acid ligase
fig|2034155.4.peg.3615	118	(3R)-hydroxymyristoyl-[ACP] dehydratase (EC 4.2.1.-)
fig|2034155.4.peg.3616	568	FIG143263: Glycosyl transferase / Lysophospholipid acyltransferase
fig|2034155.4.peg.3617	141	FIG002571: 4-hydroxybenzoyl-CoA thioesterase domain protein
fig|2034155.4.peg.3618	202	FIG027190: Putative transmembrane protein
fig|2034155.4.peg.3619	773	FIG021862: membrane protein, exporter
fig|2034155.4.peg.3620	196	FIG035331: hypothetical protein
fig|2034155.4.peg.3622	159	3-hydroxydecanoyl-[ACP] dehydratase (EC 4.2.1.59)
fig|2034155.4.peg.3654	83	Transposase
fig|2034155.4.peg.3659	192	putative thioesterase
fig|2034155.4.peg.3661	306	Permease of the drug/metabolite transporter (DMT) superfamily
fig|2034155.4.peg.3662	227	Metal-dependent hydrolase related to alanyl-tRNA synthetase
fig|2034155.4.peg.3663	314	CmaB
fig|2034155.4.peg.3666	113	Mobile element protein
fig|2034155.4.peg.3667	115	Mobile element protein
fig|2034155.4.peg.3745	260	Putative membrane protein precursor
fig|2034155.4.peg.3900	127	Phage replication protein
fig|2034155.4.peg.3995	86	Colicin immunity protein
fig|2034155.4.peg.3996	170	Translation initiation factor 2
fig|2034155.4.peg.4101	403	Mobile element protein
fig|2034155.4.peg.4104	89	Transposase, IS3/IS911 family
fig|2034155.4.peg.4161	98	Transcriptional regulator, ArsR family
fig|2034155.4.peg.4163	182	RNA polymerase sigma factor SigZ
fig|2034155.4.peg.4248	343	Phage integrase
fig|2034155.4.peg.4251	227	Phage exonuclease (EC 3.1.11.3); Putative phage-encoded enzyme involved in integration-recombination
fig|2034155.4.peg.4252	330	Recombinational DNA repair protein RecT (prophage associated)
fig|2034155.4.peg.4253	255	Oligopeptide ABC transporter, periplasmic oligopeptide-binding protein OppA (TC 3.A.1.5.1)
fig|2034155.4.peg.4255	79	Phage holin lysis protein S (ACLAME 206)
fig|2034155.4.peg.4256	171	Lysozyme (EC 3.2.1.17)
fig|2034155.4.peg.4318	109	Holin
fig|2034155.4.peg.4320	195	Chitin binding protein
fig|2034155.4.peg.4379	81	FIG01056585: hypothetical protein
fig|2034155.4.peg.4428	83	ISSod13, transposase

Table S2. J11-6^T genes that are absent in strain S32

Gene id	Length (aa)	Predicted function
fig|2034155.5.peg.144	464	DNA helicase (EC 3.6.4.12), phage-associated
fig|2034155.5.peg.146	270	Roi protein
fig|2034155.5.peg.155	182	Phage protein
fig|2034155.5.peg.156	440	Phage protein
fig|2034155.5.peg.161	220	Phage repressor
fig|2034155.5.peg.162	70	Phage Cro-like repressor protein
fig|2034155.5.peg.168	84	Probable mRNA interferase HicA
fig|2034155.5.peg.170	118	Phage holin, lambda family
fig|2034155.5.peg.175	208	Phage terminase small subunit
fig|2034155.5.peg.176	441	terminase B protein, putative
fig|2034155.5.peg.177	470	FIG01046817: hypothetical protein
fig|2034155.5.peg.178	259	FIG01047789: hypothetical protein
fig|2034155.5.peg.181	343	FIG01048949: hypothetical protein
fig|2034155.5.peg.185	314	FIG01045937: hypothetical protein
fig|2034155.5.peg.187	147	FIG01049053: hypothetical protein
fig|2034155.5.peg.190	221	FIG00644104: hypothetical protein
fig|2034155.5.peg.193	230	FIG01046179: hypothetical protein
fig|2034155.5.peg.194	115	FIG01047481: hypothetical protein
fig|2034155.5.peg.400	222	HD domain protein
fig|2034155.5.peg.674	65	DNA helicase
fig|2034155.5.peg.677	106	Mobile element protein
fig|2034155.5.peg.1080	156	Transcriptional regulator, MarR family
fig|2034155.5.peg.1110	75	Fatty acid desaturase
fig|2034155.5.peg.1187	77	4-oxalocrotonate tautomerase
fig|2034155.5.peg.1200	182	D-lyxose isomerase (EC 5.3.1.15)
fig|2034155.5.peg.1231	154	D-serine/D-alanine/glycine transporter
fig|2034155.5.peg.1424	120	Transcriptional regulator, LysR family
fig|2034155.5.peg.1426	304	Flavohemoglobin / Nitric oxide dioxygenase (EC 1.14.12.17)
fig|2034155.5.peg.1514	95	CRISPR-associated protein Cas2
fig|2034155.5.peg.1515	305	CRISPR-associated protein Cas1
fig|2034155.5.peg.1516	209	CRISPR-associated protein, Cse3 family
fig|2034155.5.peg.1517	216	CRISPR-associated protein, Cas5e family
fig|2034155.5.peg.1518	379	CRISPR-associated protein, Cse4 family
fig|2034155.5.peg.1520	509	CRISPR-associated protein, Cse1 family
fig|2034155.5.peg.1521	904	CRISPR-associated helicase Cas3
fig|2034155.5.peg.1534	692	Putative lipase
fig|2034155.5.peg.1560	230	Puative intergenic transcriptional regulator
fig|2034155.5.peg.1943	363	Prophage Lp2 protein 6
fig|2034155.5.peg.1948	585	Uncharacterized protein YfaA
fig|2034155.5.peg.1949	218	Uncharacterized protein YfaT
fig|2034155.5.peg.1951	539	Uncharacterized protein YfaQ
fig|2034155.5.peg.1952	268	Uncharacterized protein YfaP
fig|2034155.5.peg.1953	422	Putative monooxygenase

fig|2034155.5.peg.1956	147	RidA/YER057c/UK114 superfamily protein
fig|2034155.5.peg.1957	225	Protein of unknown function PA5391, DUF1028 family
fig|2034155.5.peg.1971	329	FIG01220208: hypothetical protein
fig|2034155.5.peg.1989	476	Phage integrase
fig|2034155.5.peg.1991	360	FIG00639870: hypothetical protein
fig|2034155.5.peg.1995	583	Zinc binding domain / DNA primase (EC 2.7.7.-), Phage P4-associated / Replicative helicase RepA, Phage P4-associated
fig|2034155.5.peg.1998	393	Phage major capsid protein
fig|2034155.5.peg.1999	184	Phage head maturation protease
fig|2034155.5.peg.2000	404	Phage portal protein
fig|2034155.5.peg.2001	100	FIG00639228: hypothetical protein
fig|2034155.5.peg.2002	149	Phage holin
fig|2034155.5.peg.2004	119	FIG00641869: hypothetical protein
fig|2034155.5.peg.2005	554	Phage terminase, large subunit
fig|2034155.5.peg.2072	347	Transcriptional regulator, LacI family
fig|2034155.5.peg.2073	430	Multiple sugar ABC transporter, substrate-binding protein
fig|2034155.5.peg.2077	352	FIG01423360: glycoside hydrolase
fig|2034155.5.peg.2079	373	Putative exported protein precursor
fig|2034155.5.peg.2080	425	Putative exported protein precursor
fig|2034155.5.peg.2134	101	Urease gamma subunit (EC 3.5.1.5)
fig|2034155.5.peg.2135	143	Urease beta subunit (EC 3.5.1.5)
fig|2034155.5.peg.2136	573	Urease alpha subunit (EC 3.5.1.5)
fig|2034155.5.peg.2137	207	Urease accessory protein UreE
fig|2034155.5.peg.2138	238	Urease accessory protein UreF
fig|2034155.5.peg.2140	317	Urease accessory protein UreD
fig|2034155.5.peg.2141	332	Eukaryotic-type low-affinity urea transporter
fig|2034155.5.peg.2142	353	HoxN/HupN/NixA family nickel/cobalt transporter
fig|2034155.5.peg.2145	136	late embryogenesis abundant (LEA) domain- containing protein
fig|2034155.5.peg.2156	345	alpha-L-arabinofuranosidase II (EC 3.2.1.55)
fig|2034155.5.peg.2158	169	lipoprotein, putative
fig|2034155.5.peg.2170	169	Uncharacterized protein YegL
fig|2034155.5.peg.2172	457	Integrase
fig|2034155.5.peg.2470	275	Glycosyltransferase
fig|2034155.5.peg.2477	477	Lipopolysaccharide biosynthesis protein
fig|2034155.5.peg.2492	196	Mobile element protein
fig|2034155.5.peg.2824	71	Channel-forming transporter/cytolysins activator of TpsB family
fig|2034155.5.peg.2830	439	ATP/GTP-binding protein
fig|2034155.5.peg.2831	732	ATP-dependent helicase
fig|2034155.5.peg.2832	979	Putative helicase
fig|2034155.5.peg.3093	435	FIG00775401: hypothetical protein
fig|2034155.5.peg.3095	120	RelE-like translational repressor toxin
fig|2034155.5.peg.3215	84	Octaprenyl diphosphate synthase (EC 2.5.1.90)
fig|2034155.5.peg.3301	352	Uncharacterized protein ImpA
fig|2034155.5.peg.3399	384	Putative tail fiber protein
fig|2034155.5.peg.3682	104	Mobile element protein
fig|2034155.5.peg.3683	102	Putative transposase
fig|2034155.5.peg.3707	413	COG3547: Transposase and inactivated derivatives

fig|2034155.5.peg.3984	577	Putative large exoprotein involved in heme utilization or adhesion of ShlA/HecA/FhaA family
fig|2034155.5.peg.3989	414	Putative large exoprotein involved in heme utilization or adhesion of ShlA/HecA/FhaA family
fig|2034155.5.peg.4424	206	FIG00613801: hypothetical protein
fig|2034155.5.peg.4517	413	COG3547: Transposase and inactivated derivatives
fig|2034155.5.peg.4545	75	FIG131328: Predicted ATP-dependent endonuclease of the OLD family
fig|2034155.5.peg.4561	289	Chromosome partitioning ATPase in PFGI-1-like cluster, ParA-like
fig|2034155.5.peg.4564	577	Protein with ParB-like nuclease domain in PFGI-1-like cluster
fig|2034155.5.peg.4566	190	FIG004780: hypothetical protein in PFGI-1-like cluster
fig|2034155.5.peg.4567	399	FIG141751: hypothetical protein in PFGI-1-like cluster
fig|2034155.5.peg.4568	179	Heat shock protein C
fig|2034155.5.peg.4577	811	Clamp-binding protein CrfC
fig|2034155.5.peg.4581	72	Transcriptional regulator, XRE family
fig|2034155.5.peg.4598	75	cellular communication/signal transduction
fig|2034155.5.peg.4601	91	Uncharacterized protein YrhB
fig|2034155.5.peg.4657	110	Arginine/ornithine antiporter ArcD
fig|2034155.5.peg.4661	152	FIG00208745: hypothetical protein
fig|2034155.5.peg.4666	68	Channel-forming transporter/cytolysins activator of TpsB family
fig|2034155.5.peg.4676	331	FIG00613801: hypothetical protein
fig|2034155.5.peg.4678	202	FIG00613801: hypothetical protein
fig|2034155.5.peg.4687	430	Putative large exoprotein involved in heme utilization or adhesion of ShlA/HecA/FhaA family
fig|2034155.5.peg.4698	432	Putative large exoprotein involved in heme utilization or adhesion of ShlA/HecA/FhaA family
fig|2034155.5.peg.4699	432	Putative large exoprotein involved in heme utilization or adhesion of ShlA/HecA/FhaA family
fig|2034155.5.peg.4700	423	Putative large exoprotein involved in heme utilization or adhesion of ShlA/HecA/FhaA family
fig|2034155.5.peg.4708	293	Putative large exoprotein involved in heme utilization or adhesion of ShlA/HecA/FhaA family
fig|2034155.5.peg.4709	289	Putative large exoprotein involved in heme utilization or adhesion of ShlA/HecA/FhaA family
fig|2034155.5.peg.4710	282	Putative large exoprotein involved in heme utilization or adhesion of ShlA/HecA/FhaA family
fig|2034155.5.peg.4713	219	Putative large exoprotein involved in heme utilization or adhesion of ShlA/HecA/FhaA family
fig|2034155.5.peg.4722	171	Putative large exoprotein involved in heme utilization or adhesion of ShlA/HecA/FhaA family
fig|2034155.5.peg.4723	167	FIG00613801: hypothetical protein
